# Co-occurrence of Violence-Related Risk and Protective Behaviors and Adult Support Among Male Youth in Urban Neighborhoods

**DOI:** 10.1001/jamanetworkopen.2019.11375

**Published:** 2019-09-13

**Authors:** Alison J. Culyba, Elizabeth Miller, Steven M. Albert, Kaleab Z. Abebe

**Affiliations:** 1 Division of Adolescent and Young Adult Medicine, UMPC Children’s Hospital of Pittsburgh, Pittsburgh, Pennsylvania; 2 Department of Pediatrics, University of Pittsburgh School of Medicine, Pittsburgh, Pennsylvania; 3 Department of Behavioral and Community Health Sciences, Graduate School of Public Health, University of Pittsburgh, Pittsburgh, Pennsylvania; 4 Division of General Internal Medicine, Department of Medicine, University of Pittsburgh School of Medicine, Pittsburgh, Pennsylvania

## Abstract

**Importance:**

Male youth in lower-resource neighborhoods bear a disproportionate burden of violence involvement, but little is known about clusters of specific violence-related behaviors to inform cross-cutting interventions that address multiple forms of violence.

**Objective:**

To examine associations between adult support and patterns of violence and risk or protective behavior co-occurrence among male youths in urban neighborhoods.

**Design, Setting, and Participants:**

Cross-sectional analysis of baseline and end-of-program data from a recently completed cluster-randomized sexual violence prevention trial across 20 lower-resource neighborhoods. Participants were male youths, aged 13 to 19 years, enrolled at youth-serving community agencies in Pittsburgh, Pennsylvania, from July 27, 2015, to June 5, 2017. Data were analyzed from July 1, 2018, to February 28, 2019.

**Exposures:**

Social support and natural mentoring, as defined by validated survey measures.

**Main Outcomes and Measures:**

Validated survey measures (youth violence, bullying, sexual and dating violence, history of exposure to violence and related adversities, substance use, school engagement, and future orientation) were assessed for detailed co-occurrence patterns using hierarchical clustering, dendrograms, and heatmaps across prespecified domains. Wilcoxon rank sum tests and logistic regression models examined associations between adult support and violence involvement.

**Results:**

Among 866 participants, the mean (SD) age was 15.5 (1.6) years and 632 participants (77.5%) identified as African American. All 866 participants completed baseline surveys and 577 completed end-of-program surveys. Seven clusters of risk and protective behaviors emerged: (1) school engagement; (2) career and future aspirations; (3) substance use and bullying exposure; (4) exposure to violence and related adversities, sexual violence exposure, peer delinquency, and gang involvement; (5) sexual violence, youth violence, and bullying perpetration; (6) dating abuse perpetration; and (7) physical or sexual partner violence perpetration. The strongest association cluster occurred among sexual violence perpetration behaviors. Youth with high social support engaged in significantly fewer of the 40 prespecified risk behaviors (high social support median [interquartile range], 8 [5-12] behaviors vs low social support median [interquartile range], 10 [6-14] behaviors; mean difference, 1.64 behaviors; 95% CI, 0.63-2.64 behaviors; *P *= .004). High social support and natural mentoring were both inversely associated with gang involvement (social support: odds ratio [OR], 0.39; 95% CI, 0.22-0.71; and natural mentoring: OR, 0.44; 95% CI, 0.25-0.76) and sexual violence exposure (social support: OR, 0.39; 95% CI, 0.24-0.64; and natural mentoring: OR, 0.61; 95% CI, 0.39-0.98).

**Conclusions and Relevance:**

These findings suggest that co-occurrence of risk and protective behaviors differ significantly among youth with vs without adult support. Violence prevention interventions designed to leverage adult support should address broader co-occurrence patterns.

## Key Points

**Question:**

How is adult support associated with detailed patterns of violence and risk or protective behavior co-occurrence among male youth in urban neighborhoods?

**Findings:**

In this cross-sectional study of data from a recently completed randomized clinical trial that included 866 male youths, detailed co-occurrence patterns demonstrated association clusters of sexual violence, youth violence, and bullying perpetration. Participants with social support reported significantly fewer risk behaviors.

**Meaning:**

This study suggests that violence prevention interventions designed to leverage adult support should address broader co-occurrence patterns.

## Teaser

This cross-sectional study examines associations between adult support and patterns of violence and risk and protective behavior co-occurrence among male youths in urban neighborhoods.

## Introduction

Violence is pervasive and leads to significant morbidity and mortality.^1^ Male youth in urban neighborhoods bear a disproportionate burden of individual and community violence exposure, which has been linked to increased risk of violence perpetration.^2,3,4,5,6,7,8,9,10^ Historically, violence research has tended to focus narrowly on single types of violence (eg, youth violence perpetration) and associated risk and protective factors. In 2014, the Centers for Disease Control and Prevention released a pivotal report, *Connecting the Dots: An Overview of the Links Among Multiple Forms of Violence,*^11^ which outlined interconnections among different types of violence perpetration and exposure and highlighted the urgent need for cross-cutting epidemiological and prevention studies. Understanding nuanced patterns across types of violence perpetration and associated exposures, and how these patterns align with multiple risk and protective factors among male youth in urban neighborhoods, can identify targets for intervention.

Adolescent-adult connections with family and natural mentors have shown promise in protecting youth from violence perpetration and in buffering the negative effects of violence exposure on school performance, substance use, and mental health outcomes.^12,13,14,15,16,17,18,19,20,21^ In 2014, the Centers for Disease Control and Prevention proposed promising strategies for youth violence prevention: strengthening social support and fostering adolescent-adult connections.^22^ Social support and adolescent-adult connections with family and natural mentors have also been identified as protective factors in dating violence and bullying perpetration.^23,24,25^ Research specifically among male youth in lower-resource urban neighborhoods has tended to focus on youth violence perpetration and community violence exposure, with mixed findings related to protective effects of adolescent-adult connections.^26,27,28,29,30,31,32,33^ Associations between adolescent-adult connections and other forms of violence perpetration (eg, sexual violence, bullying) are not as well characterized among youth in lower-resource urban neighborhoods.^34^ Given high rates of both violence exposure and violence perpetration in these contexts, better understanding of adolescent-adult connections and violence co-occurrence is warranted.

We used hierarchical clustering to examine associations between social support, natural mentoring, multiple forms of violence perpetration, and risk and protective behaviors among male youth in lower-resource urban neighborhoods. Our primary objective was to examine the intensity of associations across multiple violence perpetration and exposure domains and to assess how these association profiles align with adolescent-adult connections. This exploratory approach to elucidating co-occurrence patterns can help identify assets and risks that can be jointly targeted in violence prevention interventions.^34^

## Methods

### Participants and Data Source

This cross-sectional study uses data from a recently completed cluster randomized trial that enrolled 866 male adolescents aged 13 to 19 years from youth-serving community agencies in Pittsburgh, Pennsylvania, from July 27, 2015, to June 5, 2017. Eligible youths identified as male, were residents in intervention site neighborhoods, and were willing to participate in an 18-hour sexual violence prevention program. Youth could participate in the program without participating in the research. Research participants completed surveys on tablets (iPad Air; Apple) at baseline, immediately following program implementation (range of 6-15 weeks between baseline and end-of-program [EOP] survey completion), at 3 months after the intervention, and at 9 months after the intervention. Data were collected anonymously from July 27, 2015, to May 18, 2018, and linked by a participant-generated study code to encourage honest responses.^35,36^ The present analysis uses baseline and EOP data and was conducted from July 1, 2018, to February 28, 2019. All 866 participants in the program enrolled in the research study component and completed baseline surveys. A total of 577 youths completed EOP surveys that could be linked to baseline data. The University of Pittsburgh institutional review board approved the study with a waiver of parental permission. Study staff obtained verbal assent (ages 13-17 years) or consent (age ≥18 years) from youth to participate. The study design has been described in detail elsewhere.^36^ Reporting of this study follows the Strengthening the Reporting of Observational Studies in Epidemiology (STROBE) reporting guideline.

### Measuring Risk and Protective Behaviors

A total of 58 individual risk and protective behaviors were examined across prespecified domains. Baseline surveys included validated measures of youth violence perpetration (3 items), bullying perpetration (7 items), and sexual and dating violence perpetration (15 items). The EOP surveys included validated measures of bullying exposure (4 items), sexual and dating violence exposure (2 items), history of exposure to violence and related adversities (3 items), substance use (3 items), gang involvement (1 item), peer deviance (1 item), school suspension (1 item), school engagement (8 items), and career aspirations and future orientation (10 items). The term *exposure* is used to reference instances in which participants reported being the recipient of nonlethal violent behavior. Survey items are described in detail elsewhere^36^ and are summarized in eTable 1 in the Supplement. Because the primary outcome of the intervention trial from which these data emerge was sexual and dating violence perpetration, items on the baseline survey focused on this and related domains. To reduce survey burden, other domains not directly addressed through the sexual violence prevention curriculum were asked in a separate EOP survey. The time frame for reporting for each domain varied from 30 days to lifetime. Items reported with frequency scales were operationalized as 1 or more times defined as “any” and 0 times defined as “none.” Items reported with 4-point Likert scales (peer deviance) were coded as 1 for responses of 4 = “very true.” Items reported with 5-point Likert scales (school engagement, career aspirations and future orientation) were coded as 1 for responses of 4 or greater (eTable 1 in the Supplement).

### Measuring Social Support and Natural Mentoring

Participants reported on a 5-point Likert scale how often each of the following supports was available: (1) “Someone you really count on to be dependable when you need help”; (2) “Someone you really count on to care about you, regardless of what is happening to you”; and (3) “Someone you really count on to help you feel better when you are feeling down-in-the dumps” (data collected in the EOP survey).^37^ Mean responses of 4 or greater (almost all or all of the time) across the 3 items were classified as high social support. The presence of a natural mentor was defined as answering affirmatively to the question, “Is there someone at least 25 years old (but not your parent or guardian) whom you can go to for support and guidance, or if you need to make an important decision, or who inspires you to do your best?” (data collected in the EOP survey).^38^

### Statistical Analysis

To identify co-occurrence patterns across the risk and protective behavior domains, we used a method similar to the novel approach used by Ruggles and Rajan^39^ in their study of firearm carrying. To provide a comprehensive overview of participant engagement in all protective and risk behaviors, we calculated an odds ratio (OR) for each of the 3364 (58 × 58) risk and protective behavior combinations. For computational simplicity, participants’ responses to each of the 58 behaviors were analyzed as binary (see measures above). In each instance, a crude OR was calculated to provide an estimate at which the frequency of both behaviors co-occurs and is presented in an OR matrix (eTable 2 in the Supplement). In the crude OR matrix, an OR of 1 indicates no association, an OR less than 1 indicates inverse association, and an OR greater than 1 indicates direct association. To allow for OR comparisons across questions, we normalized the OR matrix using median centering and a log_2_ transformation, which aligns the data with a standard normal curve.^39^ With regard to interpretation of the normalized OR, higher positive values indicate stronger associations while lower negative values indicate weaker associations.

We used the normalized ORs to conduct hierarchical clustering, using the average distance agglomerative method, to determine which protective and risk behaviors co-occur. The primary difference between agglomerative clustering and latent class analysis is the underlying theoretical assumptions about the data, with clustering best suited for this exploratory examination of co-occurrence patterns.^40^ Output from the hierarchical clustering procedure among the entire participant sample was visualized using a dendrogram and heatmap.

To assess these co-occurrence patterns separately among youth with and without high social support, we divided the participant sample based on their social support measure and repeated the analytic procedure within each subgroup. We assessed co-occurrence patterns and mentoring in the same way. All available participant responses from baseline and EOP surveys were included in the analysis. Thus, each matrix cell included the normalized OR for all participants with data on the 2 corresponding variables (missing data <1% to 10%). All clustering analyses and visualizations were conducted using both SAS statistical software version 9.4 (SAS Institute) and R statistical software version 3.3.1 (R Project for Statistical Computing).

Informed by the dendrograms and heatmaps, we examined associations between adult support and multiple forms of violence perpetration and exposure. We first used Wilcoxon rank sum tests to compare the total number of risk and nonprotective behaviors reported by youth with vs without social support and natural mentoring using SAS version 9.4. We conducted unadjusted and adjusted (age, race/ethnicity, parental education) mixed-effects logistic regression testing the association for both (1) social support and (2) natural mentoring, with the following risk behaviors (primary dependent variables): youth violence perpetration, gang involvement, bullying perpetration, bullying exposure, sexual violence perpetration, and sexual violence exposure. Regression analyses were conducted using Stata statistical software version 14.0 (StataCorp) with 2-sided *P* < .05.

## Results

Among the 866 male participants, the mean (SD) participant age was 15.5 (1.6) years (Table 1). The majority of youths (632 participants [77.5%]) self-identified as African American and 53 youths (6.1%) self-identified as Hispanic. Most youths (734 participants [84.5%]) were currently enrolled in school, with 8th- to 11th-grade students composing most of the sample. Three hundred seventy-eight participants (43.7%) reported that their parent or guardian did not complete high school.

**Table 1.** Baseline Characteristics of Participants

Demographic Characteristics	No. (%)
No.	866
Age, y	
13-14	280 (32.3)
15-16	338 (39.0)
17-19	246 (28.4)
Race	
American Indian/Alaska Native	36 (4.4)
Asian	31 (3.8)
Black/African American	632 (77.5)
White/Caucasian	30 (3.7)
Multiracial	65 (8.0)
Other	22 (2.7)
Ethnicity	
Hispanic	53 (6.1)
Education status	
Currently in school	734 (84.8)
Grade	
8th	163 (22.2)
9th	180 (24.5)
10th	150 (20.4)
11th	130 (17.7)
12th	72 (9.8)
College	6 (0.8)
Not in school, completed high school degree	28 (3.2)
Not in school, did not complete high school	43 (4.9)
Parents’ or guardians’ highest education	
Did not complete high school	378 (43.7)
Completed high school or General Education Development testing	149 (17.2)
Attended some college	66 (7.6)
Completed college degree or higher	208 (24.0)
Violence involvement	
Physical fighting	546 (66.5)
Threatening someone with a weapon	235 (28.5)
Gang involvement	67 (12.2)
Making fun of someone or calling them hurtful names	373 (44.4)
Making threatening or aggressive comments using social media	263 (31.6)
Physically hurting dating partner	46 (5.6)
Coercing sex with dating partner	28 (3.4)

### Co-occurrence of Risk and Protective Behaviors Among the Entire Participant Sample

**Figure 1.**
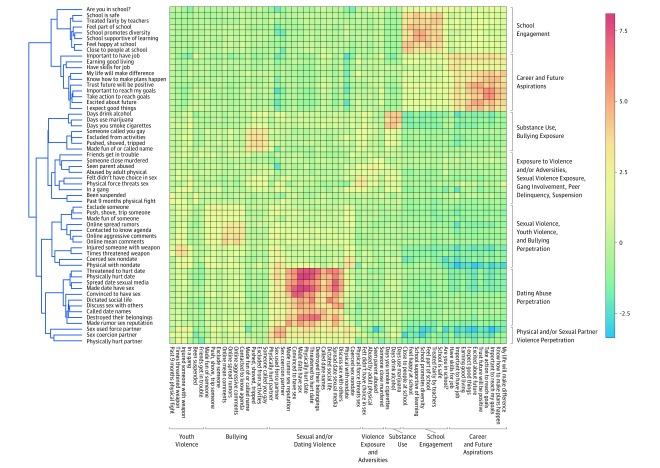
Co-occurrence of Risk and Protective Behaviors Across All Participants Dendrogram and heatmap derived from the odds ratio matrix across 58 risk and protective behaviors. The 3364 crude odds ratios were normalized and hierarchical clustering was used to define co-occurrence patterns. Color scale indicates strength of the association. The x-axis represents the prespecified domains and the y-axis represents the clustering of behaviors among participants.

Figure 1 depicts a dendrogram and heatmap that were created from the normalized OR matrix of all risk and protective behaviors across the entire participant sample. The results demonstrate the degree of similarity in the OR pattern between each of the survey items. Those behaviors that have similar associations with other behaviors are grouped most closely together. From the dendrogram, 7 overarching clusters emerged: (1) school engagement; (2) career and future aspirations; (3) substance use and bullying exposure; (4) history of exposure to violence and related adversities, sexual violence exposure, peer delinquency, and gang involvement; (5) sexual violence, youth violence, and bullying perpetration; (6) dating abuse perpetration, and (7) physical or sexual partner violence perpetration.

The heatmap depicts the relative strength of the associations across risk and protective behaviors, with the red squares demonstrating the strongest association clusters and the blue squares indicating minimal co-occurrence (Figure 1). Importantly, the normalized OR matrix represented in the heatmap does not differentiate between direct and inverse associations; directionality must be assessed through examination of the raw ORs (eTable 2 in the Supplement). The strongest association cluster occurred among sexual violence perpetration behaviors; youths who endorsed 1 of these perpetration behaviors were likely to endorse other sexual and dating violence perpetration behaviors (eg, youths who endorsed posting sexual pictures of partners had 14.0 times the odds of also reporting using physical force or threats to make someone they were going out with have sex [OR, 14.02; 95% CI, 6.16-31.93]). Sexual violence perpetration frequently co-occurred with in-person and cyberbullying perpetration and weapon-related violence perpetration. Other association clusters emerged between sexual violence exposure, bullying exposure, and history of violence exposure and related adversities (eg, youths reporting a history of coerced sex had 3.7 times the odds of being excluded from activities [OR, 3.65; 95% CI, 2.16-6.15] and 3.0 times the odds of witnessing parental abuse [OR, 3.00; 95% CI, 1.82-4.94]). Gang involvement infrequently co-occurred with violence perpetration and instead co-occurred with sexual and bullying exposure, history of exposure to violence and related adversities, and substance use (eg, participants who endorsed gang involvement had 3.5 times the odds of someone using physical force or threats to make them have sex [OR, 3.50; 95% CI, 1.83-6.71], 2.1 times the odds of parental physical abuse [OR, 2.08; 95% CI, 1.21-3.57], and 3.4 times the odds of cigarette use [OR, 3.40; 95% CI, 2.01-5.75] and marijuana use [OR, 3.40; 95% CI, 2.00-5.80]).

Future orientation and career aspirations showed similar co-occurrence patterns; youths who identified positive future orientation and strong career aspirations were unlikely to endorse sexual or weapon-related violence perpetration (eg, being excited about one’s future was associated with an OR of 0.37 [95% CI, 0.24-0.58] for physically hurting a dating partner and 0.41 [95% CI, 0.27-0.61] for injuring someone with a weapon). School engagement co-occurred with future orientation and career aspirations and was inversely associated with physical and sexual partner violence perpetration and alcohol and marijuana use. All 3364 crude ORs are available in eTable 2 in the Supplement.

### Social Support and Co-occurrence Patterns

**Figure 2.**
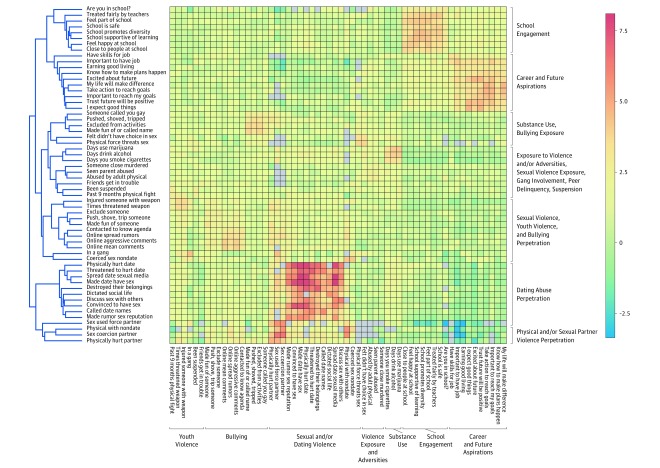
Co-occurrence of Risk and Protective Behaviors Among Youth With High Social Support Dendrogram and heatmap derived from the odds ratio matrix across 58 risk and protective behaviors among participants with high social support. The 3364 crude odds ratios were normalized and hierarchical clustering was used to define co-occurrence patterns. Color scale indicates strength of the association. The x-axis represents the prespecified domains and the y-axis represents the clustering of behaviors among participants with high social support.

**Figure 3.**
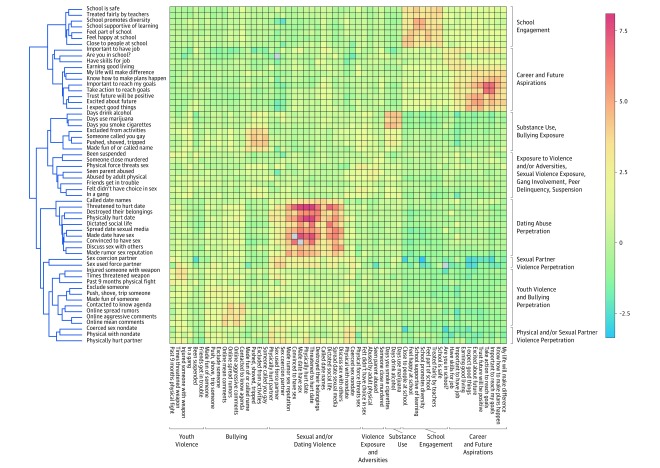
Co-occurrence of Risk and Protective Behaviors Among Youth With Low Social Support Dendrogram and heatmap derived from the odds ratio matrix across 58 risk and protective behaviors among participants with low social support. The 3364 crude odds ratios were normalized and hierarchical clustering was used to define co-occurrence patterns. Color scale indicates strength of the association. The x-axis represents the prespecified domains and the y-axis represents the clustering of behaviors among participants with low social support.

Figure 2 and Figure 3 depict the co-occurrence of risk and protective behaviors among youth with high vs low social support, respectively. Among youth with high social support, bullying exposure co-occurred most closely with sexual violence exposure. Substance use co-occurred with history of exposure to violence and related adversities, peer delinquency, school suspension, and fighting. Among youth with low social support, one key difference was that sexual and dating violence perpetration co-occurred less with other forms of violence perpetration than in the whole sample. Comparing the relative strength of the co-occurrence patterns also demonstrated distinctions between the 2 groups. Those with high social support had larger magnitudes of the direct associations between sexual violence perpetration behaviors, and between sexual violence, bullying, and youth violence perpetration. Among youth with high social support, future orientation and career aspirations showed stronger inverse associations with sexual violence perpetration, youth violence perpetration, bullying perpetration, and substance use. In contrast, among youth with low social support, school engagement was strongly inversely associated with sexual violence perpetration. In particular, feeling happy at school and attending a school that promotes diversity were inversely associated with physical and sexual partner violence perpetration and dating abuse perpetration.

### Natural Mentoring and Co-occurrence Patterns

The dendrograms depicting co-occurrence patterns among youth with vs without natural mentoring demonstrated differences in clustering of co-occurring risk and protective behaviors in the 2 groups (eFigure 1 and eFigure 2 in the Supplement). For example, among youth with natural mentoring, substance use formed its own cluster, whereas substance use was closely associated with bullying exposure among youth without natural mentoring. Sexual violence, youth violence, and bullying perpetration frequently co-occurred among youth with natural mentoring, whereas sexual violence was less closely associated with other forms of perpetration among youth without natural mentoring. Youth with natural mentoring demonstrated larger magnitudes of direct associations between multiple sexual violence perpetration behaviors.

### Quantifying Associations Between Social Support, Natural Mentoring, and Risk and Protective Behaviors

Compared with youth with low social support, youth with high social support engaged in significantly fewer of the 40 prespecified risk behaviors, with a median (interquartile range [IQR]) of 8 (5-12) behaviors in those with high social support vs a median (IQR) of 10 (6-14) behaviors among those with low social support (mean difference, 1.64 behaviors; 95% CI, 0.63-2.64 behaviors; *P* = .004). There were no significant differences in the median number of risk behaviors endorsed based on the presence of natural mentoring (median [IQR] in both groups, 9 [5-14] behaviors; mean difference, 0.67 behaviors; 95% CI, −0.38 to 1.71 behaviors; *P* = .29). Considering both the 40 risk and 18 reverse-coded protective factors jointly, youth with high social support had significantly lower exposure (median [IQR], 13 [7-18] factors) than youth with low social support (median [IQR], 17 [12-23] factors) (mean difference, 4.59 factors; 95% CI, 3.31-5.87 factors; *P* < .001). Youth with natural mentoring also had significantly lower exposure to this composite risk and reverse-coded protective factor measure (median [IQR] 15 [9-20] factors vs 17 [11-22] factors; mean difference, 1.91 factors; 95% CI, 0.55-3.27 factors; *P* = .01).

In the fully adjusted logistic regression models, high social support was inversely associated with gang involvement (OR, 0.39; 95% CI, 0.22-0.71) and sexual violence exposure (OR, 0.39; 95% CI, 0.24-0.64) (Table 2). High social support was inversely associated with youth violence perpetration in the crude model, although this association was not statistically significant after adjustment. Natural mentoring was also associated with lower odds of both gang involvement (OR, 0.44; 95% CI, 0.25-0.76) and sexual violence exposure (OR, 0.61; 95% CI, 0.39-0.98). Natural mentoring was associated with increased odds of youth violence perpetration in the fully adjusted model (OR, 1.56; 95% CI, 1.04-2.35).

**Table 2.** Associations Between Social Support, Natural Mentoring, and Multiple Forms of Violence

Form of Violence	Odds Ratio (95% CI)			
	Social Support		Natural Mentoring	
	Crude^a^	Adjusted^b^	Crude^a^	Adjusted^b^
Youth violence perpetration	0.67 (0.46-0.99)	0.74 (0.47-1.11)	1.42 (0.96-2.09)	1.56 (1.04-2.35)
Gang involvement	0.45 (0.26-0.78)	0.39 (0.22-0.71)	0.53 (0.32-0.89)	0.44 (0.25-0.76)
Bullying perpetration	0.92 (0.61-1.40)	0.80 (0.51-1.25)	1.20 (0.79-1.81)	1.11 (0.71-1.72)
Bullying exposure	0.85 (0.59-1.22)	0.81 (0.56-1.18)	0.76 (0.53-1.10)	0.80 (0.55-1.17)
Sexual violence perpetration	0.78 (0.55-1.11)	0.77 (0.53-1.12)	1.07 (0.75-1.53)	0.97 (0.66-1.41)
Sexual violence exposure	0.36 (0.22-0.58)	0.39 (0.24-0.64)	0.61 (0.39-0.94)	0.61 (0.39-0.98)

^a^ Accounting for clustering at the neighborhood level.

^b^ Additionally adjusted for age, race, caregiver education, and intervention group.

## Discussion

Among a sample of male youth in urban neighborhoods, we examined detailed co-occurrence patterns of multiple forms of violence and associated risk and protective behaviors. The strongest association cluster occurred among sexual and dating violence perpetration items, wherein youth who endorsed perpetrating 1 form of dating and sexual violence were highly likely to report perpetrating other associated behaviors. Interestingly, dating and sexual violence perpetration spanned 3 separate clusters, highlighting nuanced association patterns wherein some behaviors co-occurred more frequently and others were less closely associated. For example, making a date have sex was strongly associated with destroying their belongings, but weakly associated with coerced sex with a nondating partner. Understanding these co-occurrence patterns can inform cross-cutting prevention efforts.

In keeping with prior research, our findings highlighted how youth who perpetrate 1 form of violence (eg, youth violence) are likely to also perpetrate other forms of violence (eg, bullying).^11^ Particularly for youth in lower-resource urban settings, research has shown associations between community disadvantage, violence exposure, and multiple forms of violence perpetration.^25,30,41^ Our detailed examination of the intensity of co-occurrence patterns highlighted distinctions in the association profiles between youth violence perpetration and gang involvement, which are often considered jointly in violence research.^11^ Physical fighting was most closely associated with being suspended, whereas being in a gang was most closely associated with sexual violence exposure. In contrast, threatening or injuring someone with a weapon were strongly associated with physical and sexual violence perpetration against a nondating partner. We also noted co-occurrence of a history of exposure to violence and related adversities with sexual violence exposure, consistent with prior research examining polyvictimization.^2,11^

Examining differences in co-occurrence patterns by social support and natural mentoring provided key insights into associations across multiple forms of violence and risk and protective behaviors. Youth with high social support and youth with natural mentors reported engaging in significantly fewer risk behaviors and more protective behaviors compared with youth who lacked these supports. Both social support and natural mentoring were significantly inversely associated with gang involvement and sexual violence exposure. However, heatmaps highlighted complex associations between social support and violence co-occurrence. Among youth with high social support and among youth with a natural mentor, the association clusters of sexual violence co-occurrence were more intense and were more strongly associated with youth violence and bullying perpetration. These nuanced association profiles build on research showing that while supportive families and mentors may be associated with lower violence exposure,^26,27,28^ they may struggle to protect youth in neighborhoods with high levels of community violence.^29,30,31,32,33^ It is plausible that some adults may engage more intensively with youth already involved in violence perpetration or risk behaviors to mitigate those risks.^42^ This may in part explain the unexpected association between natural mentoring and increased odds of youth violence perpetration; youth involved in violence perpetration may reach out for support, or community-based mentors may step in to work with violence-involved youth.^11,42^ Given that social support and natural mentoring items were assessed on EOP surveys and violence perpetration was assessed on baseline surveys, caution is imperative in interpreting associations. Longitudinal studies can better define the trajectory of violence involvement and explore how mentors can best protect youth from future violence perpetration in urban neighborhoods.^18^

Inverse associations were most notable and consistent for future orientation and career aspirations, with school engagement also showing inverse associations. Among youth with high social support, future orientation and career aspirations showed stronger inverse associations with multiple sexual violence perpetration, weapon-related violence perpetration, bullying perpetration, and substance use behaviors. In contrast, among youth with low social support, school engagement was strongly inversely associated with sexual violence perpetration. Research by Stoddard and colleagues^43^ among adolescents in socially distressed urban neighborhoods found similarly complex associations between social connections, hopefulness about the future, school connectedness, and violence involvement. They found parent-family connectedness was protective against violence, and school connectedness was marginally protective. Both of these associations were mediated by hopefulness about the future. Particularly for youth who lack social support outside of school, being engaged and connected to a supportive school environment may be important in preventing sexual violence perpetration.^23,24,25^ Our cross-sectional findings support the need for longitudinal research examining the relationships between social support, school engagement, and future orientation as interconnected factors that may protect against violence perpetration.

### Limitations and Strengths

This study has some limitations. The dendrogram and heatmap approach is intended to be exploratory and hypothesis generating. Aside from Wilcoxon rank sum comparisons and logistic regression models, we did not conduct formal hypothesis testing across the more than 3000 ORs or account for multiple comparisons. Data were self-reported and time reference windows differed across some risk and protective behaviors. Social support and natural mentoring were assessed on EOP surveys, and responses may have been affected by program participation. Although this cross-sectional analysis highlighted association profiles, it did not allow us to infer temporality or causality or assess for mediation effects. Additionally, several of the sexual and dating violence perpetration items assessed similar behaviors. It is possible that the strong association cluster among these items is reflective of overlap among the items, rather than distinct experiences. The study was conducted in urban neighborhoods in a single city; results may not be generalizable to community residents who did not participate, other populations, or other geographic contexts.

The study has several important strengths. It applied innovative computational and visualization methods to examine the intensity of co-occurrence patterns across multiple forms of violence perpetration and associated exposures. The granularity afforded with this approach adds nuance to our understanding about how particular behaviors within violence domains may cluster together, and how these behaviors are associated with other risk and protective behaviors. It additionally provides key insights into the complex associations between social support and natural mentoring and violence involvement among male youth in urban neighborhoods, and highlights inverse associations between future orientation and multiple types of violence. Longitudinal studies that use additional methods (eg, latent class analysis) to examine detailed co-occurrence profiles over time may help elucidate causal mechanisms underpinning the observed associations.

## Conclusions

In this detailed examination of co-occurrence patterns across key violence perpetration and exposure domains and important risk and protective behaviors, we identified inverse associations between both social support and natural mentoring, and associations between these supports, future orientation, and school engagement. Results can encourage development of violence prevention programs that recognize complex co-occurrence and address multiple forms of violence simultaneously.^11^ Such integrated approaches can make expeditious use of limited resources and increase the public health impact of violence prevention programs.

## Notes

Supplement. **eTable 1.** Measures of Violence, Risk and Protective Behaviors **eTable 2.** Matrix of Crude Odds Ratios of Associations Across the 3364 Risk and Protective Behaviors Among All Study Participants **eFigure 1.** Co-occurrence of Risk and Protective Behaviors Among Youth With Natural Mentoring **eFigure 2.** Co-occurrence of Risk and Protective Behaviors Among Youth Without Natural Mentoring
