# Supplementary material for: Co-occurrence of Violence-Related Risk and Protective Behaviors and Adult Support Among Male Youth in Urban Neighborhoods
Source: JAMA Netw Open. 2019 Sep 13;2(9):e1911375. doi: 10.1001/jamanetworkopen.2019.11375 (PMC6745057; doi:10.1001/jamanetworkopen.2019.11375)
Supplement: Supplement. — eTable 1. Measures of Violence, Risk and Protective Behaviors eTable 2. Matrix of Crude Odds Ratios of Associations Across the 3364 Risk and Protective Behaviors Among All Study Participants eFigure 1. Co-occurrence of Risk and Protective Behaviors Among Youth With Natural Mentoring eFigure 2. Co-occurrence of Risk and Protective Behaviors Among Youth Without Natural Mentoring [file jamanetwopen-2-e1911375-s001.pdf]

Supplementary Online Content

Culyba AJ, Miller E, Albert SM, Abebe KZ. Co-occurrence of violence-related risk and protective behaviors and adult support among male youth in urban neighborhoods. *JAMA Netw Open*. 2019;2(9):e1911375. doi:10.1001/jamanetworkopen.2019.11375

eTable 1. Measures of Violence, Risk and Protective Behaviors

eTable 2. Matrix of Crude Odds Ratios of Associations Across the 3364 Risk and Protective Behaviors Among All Study Participants

eFigure 1. Co-occurrence of Risk and Protective Behaviors Among Youth With Natural Mentoring

eFigure 2. Co-occurrence of Risk and Protective Behaviors Among Youth Without Natural Mentoring

This supplementary material has been provided by the authors to give readers additional information about their work.

© 2019 Culyba AJ et al. *JAMA Network Open.*

eTable 1: Measures of violence, risk and protective behaviors

Domain	Survey		Reporting period	Response options & procedure to dichotomize	Questions
	Baseline	End of Program			
Youth violence perpetration	x		9 months	5 category response:<br>-- 0 times<br>--1 time<br>--2 to 4 times<br>--5 to 9 times<br>--10 or more times<br>(Cronbach's alpha 0.58)<br><br>Modeled as yes to 1 time or more	In the past 9 months, how many times…<br>1. Were you in a physical fight? (n=821)<br>2. Have you threatened to injure someone with a weapon such as a gun, knife, or club? (n=826)<br>3. Have you physically injured someone with a weapon such as a gun, knife, or club? (n=826)
Bullying perpetration	x		3 months	4-point scale for frequency:<br>--Never<br>--A few times<br>--Once or twice a week<br>--Every day or almost every day<br>(Cronbach's alpha 0.68)<br><br>Modeled as yes for any response of 'a few times' or more often	*In the past 3 months, how often did you do the following to someone? How often did you…*<br>1. Make fun of someone or called them names in a hurtful way, or spread rumors about them? (n=840)<br>2. Push, shove, trip, or spit on someone? (n=843)<br>3. Exclude someone from activities on purpose? (n=837)
Cyberbullying perpetration	x		3 months	4-point scale for frequency:<br>--Never<br>--A few times<br>--Once or twice a week<br>--Every day or almost every day<br>(Cronbach's alpha 0.79)<br><br>Modeled as yes for any response of 'a few times' or more often	*In the past 3 months, how often did you do the following to someone? How often did you…*<br>1. …make mean or hurtful comments… (n=834)<br>2. …spread rumors… (n=834)<br>3. …make threatening or aggressive comments…(n=833)<br>4. …repeatedly contact them to see where they were and/or who they were with…(n=831)<br>… using mobile apps, social networks, texts, or other digital communication?
Bullying exposure		x	3 months	4-point scale for frequency:<br>--Never<br>--A few times<br>--Once or twice a week<br>--Every day or almost every day<br>(Cronbach's alpha 0.83)<br><br>Modeled as yes for any response of 'a few times' or more often	*In the past 3 months, how often has someone done the following to you? How often did someone…*<br>1. Make fun of you or called you names in a hurtful way, or spread rumors about you? (n=546)<br>2. Pushed, shoved, tripped, or spit on you? (n=542)<br>3. Excluded you from activities on purpose? (n=536)<br>4. Some kids call each other names like homo or gay. How many times in the past 3 months has someone called YOU homo or gay? (n=540)_

© 2019 Culyba AJ et al. *JAMA Network Open.*

Physical/sexual partner violence perpetration	x		Lifetime	Yes/no<br>(Cronbach's alpha 0.62; KR-20 0.63)	*Have YOU done any of the following to someone you were in a relationship with (like he or she was your partner/girlfriend/ boyfriend, you were dating or going out with them) or hooking up with:*<br>1. …hit, pushed, slapped, choked or otherwise physically hurt someone you were going out with or hooking up with? (include such things as hitting, slamming into something, or injuring with an object or weapon.) (n=826)<br>2. …used physical force or threats to make someone you were going out with or hooking up with have sex (vaginal, oral, or anal sex) when they didn't want to? (n=838)<br>3. …had sex with someone you were going out with or hooking up with when they didn't want to or because you made them feel like they didn't have a choice (even though you did not use physical force or threats)? (n=826)
Dating abuse perpetration	x		Lifetime	Yes/no<br>(Cronbach's alpha 0.89)	*Have YOU done any of the following to someone you were going out with (like he or she was your partner/girlfriend/boyfriend, you were dating them) or hooking up with:*<br>1. Spread rumors about their sexual reputation, like telling people they're 'easy'. (n=823)<br>2. Convinced them to have sex, after they had said no a few times. (n=819)<br>3. Made them have sex when they didn't want to. (n=822)<br>4. Physically hurt them (like shoving, grabbing, slapping, punching, choking). (n=821)<br>5. Threatened to hurt them if they didn't do what you wanted them to do. (n=820)<br>6. Yelled at them or destroyed something that belonged to them. (n=818)<br>7. Called them names, like ugly or stupid. (n=813)<br>8. Told them not to talk to others or told them who they could hang out with. (n=817)<br>9. Showed friends or posted pictures of them naked or doing something sexual. (n=819)<br>10. Talked about what you and your partner do sexually with your friends or peers. (n=818)
Non-partner sexual violence perpetration	x		Lifetime	Yes/no<br>(Cronbach's alpha 0.47; KR-20 0.47)	*Now think about experiences you may have had with people who you were NOT going out with or hooking up with (this could include strangers, friends, family, or people you don't know well).*<br><br>*Please tell us whether YOU have ever done these things to anyone you were NOT going out or hooking up with:*<br>1. …used physical force or threats to make someone you were not going out with or hooking up with have sex (vaginal, oral, or anal sex) with you when they didn't want to? (n=834)

© 2019 Culyba AJ et al. *JAMA Network Open.*

					2. …insisted that someone you were not going out with or hooking up with have sex (vaginal, oral, or anal sex) when they didn't want to, without using force or threats? (n=827)
Sexual violence exposure		x	Lifetime	Yes/no<br>(Cronbach's alpha 0.54; KR-20 0.54)	Now we want to ask you about things that someone may have done to you ("Someone" could be someone you were going out with or hooking up with, a person you know, a friend, a family member, or a stranger):<br>1. Has someone ever used physical force or threats to make you have sex (vaginal, oral, or anal sex) when you didn't want to? (n=546)<br>2. Have you ever had sex with someone when you didn't want to, because you felt like you didn't have a choice, even though they did not use physical force or threats? (n=544)
Exposure to violence and related adversities		x	Lifetime	Yes/no<br>(Cronbach's alpha 0.47; KR-20 0.47)	1. Not including spanking on your bottom, at any time in your life did a grown-up in your life hit, beat, kick or physically hurt you in any way? (n=543)<br>2. At any time in your life did you see a parent get pushed, slapped, hit, punched or beat up by another parent, or by his or her boyfriend or girlfriend? (n=541)<br>3. At any time in your life, was anyone close to you murdered, like a friend, neighbor, or someone in your family? (n=541)
Substance use		x	30 days	7 category response:<br>-- 0 days<br>-- 1 or 2 days<br>-- 3 to 5 days<br>-- 6 to 9 days<br>-- 10 to 19 days<br>-- 20 to 29 days<br>-- All 30 days<br>(Cronbach's alpha 0.76)<br><br>Modeled as yes to one day or more	During the past 30 days, on how many days…<br>1. did you smoke cigarettes (or other tobacco products)? (n=557)<br>2. did you have at least one drink of alcohol? (n=551)<br>3. did you use marijuana? (n=549)
Gang involvement		x		Yes/no	1. Do you consider yourself a member of a gang? (n=550)
Peer deviance		x		4-point Likert scale from 'not at all true' to 'very true'<br><br>Modeled as yes for 4='very true'	2. How true is the following statement: My friends get into a lot of trouble. (n=555)
School suspension		x	Lifetime	Yes/no	3. Have you ever been suspended from school? (n=551)
School connectedness/ engagement		x		5-point Likert scale from 'strongly disagree' to 'strongly agree'<br>(Cronbach's alpha 0.92)	*Please select how you feel about the following statements.*<br>1. I feel close to people at my school. (n=505)<br>2. I feel happy at my school. (n=503)

© 2019 Culyba AJ et al. *JAMA Network Open.*

				Modeled as yes for 5='strongly agree' and 4='somewhat agree'	3. My school is a supportive and inviting place for students to learn. (n=495)<br>4. My school fosters an appreciation of student diversity and respect for each other. (n=501)<br>5. I feel like I am part of my school. (n=501)<br>6. I feel teachers at my school treat students fairly. (n=497)<br>7. My school is a safe place for students (n=497)<br>*Items only asked of those participants in school.*<br><br>*8.* Are you in school? (n=564)
Career aspirations	x			Item 1: 5-point Likert scale from 'strongly disagree' to 'strongly agree'<br>Item 2: 5-point Likert scale from 'not at all Important' to 'very important'<br>Item 3: 5-point Likert scale from 'extremely poor' to 'excellent'<br>(Cronbach's alpha 0.74)<br><br>Modeled as yes for ≥4	1. I have enough skills to do a job well. (n=806)<br>2. How important is it to you to have a good job or career? (n=807)<br>3. What do you think your chances are of earning a good living? (n=809)
Future orientation	x			5-point Likert scale from 'not at all like me' to 'exactly like me,' (Cronbach's alpha 0.93)<br><br>Modeled as yes for 5='exactly like me' and 4= 'somewhat like me'	1. I expect good things to happen to me. (n=817)<br>2. I am excited about my future. (n=816)<br>3. I trust my future will turn out well. (n=817)<br>4. If I set goals, I take action to reach them. (n=813)<br>5. It is important to me that I reach my goals. (n=815)<br>6. I know how to make my plans happen. (n=815)<br>7. My life will make a difference in the world. (n=809)
Social supports		x		5-point Likert scale from 'none of the time' to 'all of the time,' (Cronbach's alpha 0.87)<br><br>Modeled as yes for mean response across the 3 items of ≥4	*The following questions ask about people who provide you with help or support. How often is each of the following supports available to you when you need it?*<br>1. Someone you really count on to be dependable when you need help. (n=531)<br>2. Someone you really count on to care about you, regardless of what is happening to you. (n=528)<br>3. Someone you really count on to help you feel better when you are feeling generally down-in-the-dumps. (n=518)
Natural mentors		x		Yes/no	1. Is there someone at least 25 years old (but NOT your parent or guardian) whom you can go to for support and guidance, or if you need to make an important decision, or who inspires you to do your best? (n=554)

KR-20 refers to Kuder-Richardson coefficient of reliability

© 2019 Culyba AJ et al. *JAMA Network Open.*

Supplemental Table 2: Matrix of Crude Odds Ratios of Associations across the 3,364 risk and protective behaviors among all study participants

	q1	q2	q3	q4	q5	q6	q7	q8	q9	q10	q11	q12	q13	q14	q15	q16	q17	q18	q19	q20	q21	q22	q23	q24	q25	q26	q27	q28	q29	q30	q31	q32	q33	q34	q35	q36	q37	q38	q39	q40	q41	q42	q43	q44	q45	q46	q47	q48	q49	q50	q51	q52	q53	q54	q55	q56	q57	q58
q1 = PHYSICALLY_HURT_PARTNER		14.99	18.42	4.55	4.63	3.84	9.75	3.95	5.04	4.68	4.86	3.07	4.76	6.13	4.14	2.34	2.63	3.70	3.73	3.03	3.37	2.95	1.86	5.35	5.98	1.70	0.85	0.51	1.11	0.79	0.91	1.30	1.07	0.69	1.46	2.60	2.73	1.46	1.96	2.03	1.19	0.65	1.25	1.73	1.26	0.75	0.55	0.84	0.78	0.80	1.11	0.82	0.47	0.91	0.94	0.78	0.84	0.50
q2 = SEX_USED_FORCE_PARTNER	14.99		44.97	10.99	10.26	11.97	21.86	19.58	9.80	6.43	11.55	14.02	4.57	19.18	7.69	2.44	4.21	5.52	3.56	1.73	5.33	2.96	1.53	8.24	7.41	4.76	0.25	0.20	0.40	0.35	0.17	0.31	0.48	2.91	1.21	0.45	1.93	1.45	1.65	0.84	0.23	0.52	1.75	2.98	1.97	0.64	0.45	0.58	0.24	0.27	0.45	1.13	1.12	0.72	0.55	0.25	0.36	0.27
q3 = SEX_COERCION_PARTNER	18.42	44.97		6.95	7.24	10.52	9.61	7.75	5.46	3.88	6.25	6.60	4.22	9.68	6.03	1.66	3.08	2.56	3.20	1.26	3.00	2.42	3.55	3.15	3.82	3.32	0.37	0.39	0.54	0.45	0.35	0.82	0.82	2.18	2.71	1.59	1.54	1.61	1.48	0.94	0.92	1.48	1.59	3.59	2.69	0.26	0.55	0.41	0.24	0.49	0.80	0.55	0.92	0.48	0.87	0.37	0.53	0.52
q4 = MADE_RUMOR_SEX_REPUTATION	4.55	10.99	6.95		30.88	78.93	40.92	49.36	25.71	15.56	15.46	51.33	18.19	3.34	2.59	3.24	3.50	3.34	2.56	2.80	2.99	2.36	2.41	2.85	1.91	2.35	0.65	0.63	0.93	0.85	0.73	0.80	0.67	0.69	2.67	1.11	1.29	1.28	1.45	1.59	1.35	1.36	1.87	1.84	2.28	0.95	0.74	0.76	0.70	0.69	0.61	0.93	0.89	1.49	0.75	0.77	0.67	0.83
q5 = CONVINCED_TO_HAVE_SEX	4.63	10.26	7.24	30.88		245.26	72.62	80.03	25.05	14.90	25.98	72.52	28.00	3.07	2.11	2.37	2.51	3.69	3.16	2.41	3.31	2.02	1.61	2.37	2.30	1.56	0.78	0.64	0.90	0.77	0.70	0.82	0.71	1.15	1.53	0.96	1.16	1.36	0.86	1.33	0.77	1.62	1.34	1.49	1.86	0.84	0.71	0.90	0.71	0.60	0.66	0.87	1.21	1.66	0.74	0.94	0.82	1.01
q6 = MADE_DATE_HAVE_SEX	3.84	11.97	10.52	78.93	245.26		433.20	266.20	110.50	31.40	62.38	231.06	57.71	4.14	2.38	2.35	2.69	2.47	2.48	2.32	2.26	1.64	1.56	2.28	2.48	1.71	0.46	0.39	0.61	0.54	0.47	0.57	0.55	1.57	1.71	0.86	1.37	1.29	0.95	1.21	0.93	1.27	1.86	2.35	2.23	0.96	1.02	0.84	0.67	0.69	0.84	0.97	1.09	1.04	0.52	0.50	0.80	0.83
q7 = PHYSICALLY_HURT_DATE	9.75	21.86	9.61	40.92	72.62	433.20		302.32	115.82	36.69	40.22	125.66	36.28	5.26	2.40	2.26	2.23	2.91	2.48	1.78	2.42	1.59	1.84	2.87	2.74	1.19	0.53	0.37	0.81	0.51	0.42	0.62	0.49	1.15	1.78	0.94	1.49	1.10	0.91	1.33	1.56	1.04	1.76	2.01	1.99	1.19	0.92	1.01	0.92	0.89	1.02	1.13	0.72	0.82	0.48	0.55	0.68	0.75
q8 = THREATENED_TO_HURT_DATE	3.95	19.58	7.75	49.36	80.03	266.20	302.32		172.50	41.88	103.37	100.06	43.48	3.41	1.60	2.31	2.39	2.08	2.46	1.80	2.20	1.28	1.75	2.56	2.38	1.31	0.46	0.36	0.67	0.51	0.39	0.50	0.62	1.22	1.96	0.79	1.68	1.43	1.18	0.83	0.90	0.96	1.68	2.05	1.88	1.42	1.07	1.20	0.90	1.04	0.88	1.18	0.60	0.83	0.51	0.53	0.59	0.74
q9 = DESTROYED_THEIR_BELONGINGS	5.04	9.80	5.46	25.71	25.05	110.50	115.82	172.50		30.84	20.75	55.03	20.31	2.94	1.40	2.59	2.20	2.49	3.32	2.09	1.91	1.49	1.89	2.39	2.08	1.17	0.75	0.51	1.09	0.69	0.68	0.75	0.67	0.98	1.48	1.60	1.19	1.52	1.00	1.30	0.99	1.43	1.79	1.86	1.93	0.88	0.86	0.64	0.72	0.63	0.85	0.94	0.98	1.17	0.95	0.92	0.85	1.24
q10 = CALLED_DATE_NAMES	4.68	6.43	3.88	15.56	14.90	31.40	36.69	41.88	30.84		14.20	19.84	11.12	1.40	1.50	2.29	2.12	2.41	2.28	2.43	2.44	1.67	2.17	2.21	1.82	1.30	1.09	0.95	1.60	1.07	1.40	1.25	0.86	0.65	1.66	1.67	1.48	1.51	1.29	1.06	1.18	1.58	2.42	1.69	2.28	1.49	1.06	1.07	1.00	0.98	0.87	1.04	0.79	1.56	1.07	1.57	1.17	1.48
q11 = DICTATED_SOCIAL_LIFE	4.86	11.55	6.25	15.46	25.98	62.38	40.22	103.37	20.75	14.20		41.39	16.65	2.31	1.79	2.52	2.19	2.40	2.93	2.08	1.81	1.62	1.61	2.23	2.43	0.82	0.84	0.79	1.20	1.05	1.00	1.15	0.80	0.83	1.62	1.11	0.93	0.94	1.08	1.25	1.07	1.14	1.74	1.76	2.26	0.95	0.78	0.87	0.85	1.00	0.98	1.11	0.68	1.25	0.88	1.40	0.99	1.08
q12 = SPREAD_DATE_SEXUAL_MEDIA	3.07	14.02	6.60	51.33	72.52	231.06	125.66	100.06	55.03	19.84	41.39		48.16	4.27	2.69	2.36	2.59	2.57	2.18	1.89	2.57	1.64	1.36	2.32	3.03	1.71	0.55	0.39	0.71	0.49	0.45	0.57	0.58	0.82	1.36	0.97	1.73	1.27	1.19	1.03	1.11	0.93	2.16	2.26	2.12	1.06	0.82	0.76	0.71	0.62	0.86	1.02	0.71	0.87	0.55	0.58	0.71	0.95
q13 = DISCUSS_SEX_WITH_OTHERS	4.76	4.57	4.22	18.19	28.00	57.71	36.28	43.48	20.31	11.12	16.65	48.16		1.64	2.22	2.53	2.18	2.57	2.10	2.47	1.81	1.82	1.79	1.97	2.28	1.37	0.84	0.70	1.03	0.75	0.83	1.08	0.83	0.58	1.46	1.33	1.38	1.77	1.29	1.24	0.90	1.51	1.93	1.93	2.04	0.84	0.86	0.80	0.80	0.72	0.81	0.73	0.71	1.20	1.08	1.05	0.85	0.99
q14 = PHYSICAL_WITH NON_DATE	6.13	19.18	9.68	3.34	3.07	4.14	5.26	3.41	2.94	1.40	2.31	4.27	1.64		14.74	2.99	3.38	3.29	2.61	4.71	3.83	4.33	1.53	4.05	5.49	3.94	0.32	0.17	0.25	0.41	0.23	0.37	0.24	0.48	2.78	1.47	1.96	2.99	1.29	1.53	1.91	0.93	1.59	1.44	1.80	0.79	0.77	1.19	1.33	1.14	0.82	0.65	0.77	1.34	0.32	0.18	0.27	0.43
q15 = COERCED_SEX NON_DATE	4.14	7.69	6.03	2.59	2.11	2.38	2.40	1.60	1.40	1.50	1.79	2.69	2.22	14.74		1.97	3.84	2.42	2.38	3.83	3.59	2.44	1.41	3.48	5.25	4.04	0.57	0.73	0.61	0.83	0.69	0.85	0.74	1.89	1.75	1.26	1.16	1.74	1.22	2.49	1.32	1.25	2.36	2.16	2.52	0.42	0.52	0.80	0.71	0.89	0.68	0.65	2.06	1.11	0.57	0.35	0.55	0.31
q16 = ONLINE_MEAN_COMMENTS	2.34	2.44	1.66	3.24	2.37	2.35	2.26	2.31	2.59	2.29	2.52	2.36	2.53	2.99	1.97		7.70	19.08	9.69	4.40	4.19	3.11	1.85	3.25	3.06	1.06	0.85	0.93	0.77	0.79	0.85	0.85	0.80	0.79	1.03	1.53	1.31	1.42	1.31	1.09	1.14	1.14	1.07	1.03	1.11	1.26	1.14	1.04	1.12	1.18	0.60	0.86	1.02	0.97	1.21	0.80	0.86	0.99
q17 = ONLINE_SPREAD_RUMORS	2.63	4.21	3.08	3.50	2.51	2.69	2.23	2.39	2.20	2.12	2.19	2.59	2.18	3.38	3.84	7.70		12.25	14.76	4.80	4.39	4.78	1.57	2.97	4.20	1.45	0.65	0.62	0.59	0.67	0.58	0.85	0.78	1.12	1.76	2.00	1.88	2.31	2.23	1.39	0.94	1.09	1.24	1.74	1.33	1.11	1.14	1.36	1.02	1.10	0.74	1.01	1.51	0.98	0.70	0.37	0.86	0.82
q18 = ONLINE_AGRESSIVE_COMMENTS	3.70	5.52	2.56	3.34	3.69	2.47	2.91	2.08	2.49	2.41	2.40	2.57	2.57	3.29	2.42	19.08	12.25		11.24	4.34	5.15	3.82	1.67	4.11	3.64	1.31	0.73	0.67	0.72	0.69	0.71	0.79	0.69	0.70	1.06	1.50	1.42	1.40	1.49	1.18	1.57	1.20	1.16	1.17	1.47	0.83	0.80	0.97	0.81	1.12	0.65	0.80	1.57	1.32	1.01	0.59	0.76	0.98
q19 = CONTACTED_TO_KNOW_AGENDA	3.73	3.56	3.20	2.56	3.16	2.48	2.48	2.46	3.32	2.28	2.93	2.18	2.10	2.61	2.38	9.69	14.76	11.24		3.07	4.19	3.85	1.64	3.51	3.34	1.22	0.68	0.69	0.78	0.84	0.77	0.92	0.95	1.07	1.70	1.59	1.25	1.67	1.29	1.18	1.31	1.22	1.41	1.45	1.34	1.29	1.33	1.31	1.05	1.29	0.89	1.17	1.53	1.40	0.87	0.56	0.74	1.17
q20 = MADE_FUN_OF_SOMEONE	3.03	1.73	1.26	2.80	2.41	2.32	1.78	1.80	2.09	2.43	2.08	1.89	2.47	4.71	3.83	4.40	4.80	4.34	3.07		4.27	4.81	1.77	2.70	2.51	1.87	0.87	0.77	0.86	1.03	1.17	0.95	0.88	1.03	1.81	1.83	1.85	1.64	2.01	1.65	1.60	1.26	1.10	1.20	1.15	0.95	1.01	0.96	0.96	1.10	0.78	0.65	1.64	1.20	1.00	0.85	0.63	0.81
q21 = PUSH_SHOVE_TRIP_SOMEONE	3.37	5.33	3.00	2.99	3.31	2.26	2.42	2.20	1.91	2.44	1.81	2.57	1.81	3.83	3.59	4.19	4.39	5.15	4.19	4.27		5.72	2.15	3.22	3.98	2.22	0.82	0.73	0.85	0.72	0.87	0.79	0.67	1.11	1.81	1.34	1.56	1.31	1.61	1.38	1.71	1.26	1.27	1.51	1.39	1.04	0.71	0.89	0.82	0.90	0.56	0.75	1.80	1.29	0.70	0.54	0.80	0.93
q22 = EXCLUDE_SOMEONE	2.95	2.96	2.42	2.36	2.02	1.64	1.59	1.28	1.49	1.67	1.62	1.64	1.82	4.33	2.44	3.11	4.78	3.82	3.85	4.81	5.72		1.69	2.85	3.10	2.49	0.75	0.67	0.95	0.68	0.78	1.06	0.69	1.21	1.33	1.32	2.03	1.88	1.61	1.48	1.56	1.05	1.31	1.37	1.68	0.76	0.71	0.80	0.93	0.80	0.65	0.77	1.17	1.30	0.81	0.58	0.62	0.98
q23 = PAST_9_MTHS_PHYS_FIGHT	1.86	1.53	3.55	2.41	1.61	1.56	1.84	1.75	1.89	2.17	1.61	1.36	1.79	1.53	1.41	1.85	1.57	1.67	1.64	1.77	2.15	1.69		3.83	3.45	1.59	0.81	0.88	0.82	0.97	0.95	0.98	0.77	1.24	1.85	1.40	1.24	1.81	1.44	1.26	1.34	1.46	1.79	1.59	2.14	0.85	0.71	0.82	0.89	0.94	0.63	0.69	1.54	3.97	1.23	1.10	0.83	1.02
q24 = TIMES_THREATENED_WEAPON	5.35	8.24	3.15	2.85	2.37	2.28	2.87	2.56	2.39	2.21	2.23	2.32	1.97	4.05	3.48	3.25	2.97	4.11	3.51	2.70	3.22	2.85	3.83		22.60	3.28	0.68	0.68	0.64	0.78	0.63	0.87	0.73	0.97	3.01	1.69	1.43	1.73	1.25	2.28	1.56	1.38	1.80	1.29	1.75	0.76	0.77	0.75	0.71	0.80	0.65	0.75	1.47	1.87	0.73	0.55	0.72	1.15
GR25 = INJURED_SOMEONE_WITH_WEAPON	5.98	7.41	3.82	1.91	2.30	2.48	2.74	2.38	2.08	1.82	2.43	3.03	2.28	5.49	5.25	3.06	4.20	3.64	3.34	2.51	3.98	3.10	3.45	22.60		4.68	0.54	0.41	0.55	0.45	0.41	0.73	0.55	1.58	1.97	2.46	1.73	2.68	1.68	1.87	1.37	1.74	1.96	2.14	1.56	0.75	0.75	0.81	0.68	0.68	0.61	0.68	1.80	1.66	0.58	0.35	0.66	0.63
q26 = IN_A_GANG	1.70	4.76	3.32	2.35	1.56	1.71	1.19	1.31	1.17	1.30	0.82	1.71	1.37	3.94	4.04	1.06	1.45	1.31	1.22	1.87	2.22	2.49	1.59	3.28	4.68		1.21	0.91	0.58	0.89	0.78	1.00	1.24	3.50	4.50	1.77	2.68	2.86	2.30	2.08	1.55	0.99	3.40	2.60	3.40	0.60	0.60	0.85	0.79	0.84	0.56	0.70	1.86	1.26	0.88	0.30	1.05	0.42
q27 = I_EXPECT_GOOD_THINGS	0.85	0.25	0.37	0.65	0.78	0.46	0.53	0.46	0.75	1.09	0.84	0.55	0.84	0.32	0.57	0.85	0.65	0.73	0.68	0.87	0.82	0.75	0.81	0.68	0.54	1.21		15.54	20.44	10.91	17.10	8.31	7.36	1.25	0.77	0.97	0.67	0.77	0.87	0.98	0.80	0.96	0.50	0.55	0.68	1.65	1.81	1.83	1.52	1.53	1.26	1.56	1.17	1.21	2.63	8.59	3.98	1.28
q28 = EXCITED_ABOUT_FUTURE	0.51	0.20	0.39	0.63	0.64	0.39	0.37	0.36	0.51	0.95	0.79	0.39	0.70	0.17	0.73	0.93	0.62	0.67	0.69	0.77	0.73	0.67	0.88	0.68	0.41	0.91	15.54		31.68	15.60	20.58	11.72	12.32	1.42	0.78	0.94	0.74	0.83	0.86	0.88	0.93	1.58	0.50	0.46	0.57	2.00	1.96	2.56	1.87	2.48	1.81	2.36	0.90	1.19	3.16	9.11	5.75	2.01
q29 = TRUST_FUTURE_WILL_BE_POSITIVE	1.11	0.40	0.54	0.93	0.90	0.61	0.81	0.67	1.09	1.60	1.20	0.71	1.03	0.25	0.61	0.77	0.59	0.72	0.78	0.86	0.85	0.95	0.82	0.64	0.55	0.58	20.44	31.68		15.73	24.89	14.04	13.46	0.87	0.65	1.10	0.88	0.81	1.13	0.93	1.23	1.25	0.60	0.53	0.67	2.53	2.46	2.60	2.11	2.56	2.10	2.26	0.92	1.01	2.89	9.48	5.94	2.02
q30 = TAKE_ACTION_TO_REACH_GOALS	0.79	0.35	0.45	0.85	0.77	0.54	0.51	0.51	0.69	1.07	1.05	0.49	0.75	0.41	0.83	0.79	0.67	0.69	0.84	1.03	0.72	0.68	0.97	0.78	0.45	0.89	10.91	15.60	15.73		52.89	20.10	14.37	1.12	0.92	1.04	0.75	0.66	1.02	1.16	0.80	1.44	0.70	0.59	0.72	1.85	1.69	1.97	1.51	1.96	1.87	1.57	0.98	1.01	3.42	9.24	5.04	2.03
q31 = IMPORTANT_TO_REACH_MY_GOALS	0.91	0.17	0.35	0.73	0.70	0.47	0.42	0.39	0.68	1.40	1.00	0.45	0.83	0.23	0.69	0.85	0.58	0.71	0.77	1.17	0.87	0.78	0.95	0.63	0.41	0.78	17.10	20.58	24.89	52.89		22.95	17.37	0.94	0.91	1.63	0.98	0.88	1.10	0.95	0.83	1.89	0.58	0.40	0.54	1.85	1.92	2.22	1.34	1.67	1.88	2.03	1.02	1.34	3.92	11.86	4.60	2.78
q32 = KNOW_HOW_TO_MAKE_PLANS_HAPPEN	1.30	0.31	0.82	0.80	0.82	0.57	0.62	0.50	0.75	1.25	1.15	0.57	1.08	0.37	0.85	0.85	0.85	0.79	0.92	0.95	0.79	1.06	0.98	0.87	0.73	1.00	8.31	11.72	14.04	20.10	22.95		11.34	1.09	1.07	0.84	0.81	0.80	0.88	1.20	1.10	1.38	0.84	0.67	0.99	1.75	1.80	1.55	1.51	1.77	1.59	1.54	0.71	0.78	3.23	7.50	4.71	2.16
q33 = MY_LIFE_WILL_MAKE_DIFFERENCE	1.07	0.48	0.82	0.67	0.71	0.55	0.49	0.62	0.67	0.86	0.80	0.58	0.83	0.24	0.74	0.80	0.78	0.69	0.95	0.88	0.67	0.69	0.77	0.73	0.55	1.24	7.36	12.32	13.46	14.37	17.37	11.34		2.11	1.03	1.51	0.81	1.09	1.06	1.05	0.82	1.24	0.74	0.84	0.77	1.59	1.74	2.13	1.24	1.80	1.56	1.76	0.91	0.83	2.60	5.87	5.37	2.11
q34 = PHYSICAL_FORCE_THREATS_SEX	0.69	2.91	2.18	0.69	1.15	1.57	1.15	1.22	0.98	0.65	0.83	0.82	0.58	0.48	1.89	0.79	1.12	0.70	1.07	1.03	1.11	1.21	1.24	0.97	1.58	3.50	1.25	1.42	0.87	1.12	0.94	1.09	2.11		9.75	2.34	2.41	2.81	2.73	2.44	3.71	1.27	1.89	2.25	1.85	0.85	0.77	0.93	0.86	0.71	0.88	1.17	1.44	1.45	0.92	1.27	2.06	1.02
q35 = FELT_DIDNT_HAVE_CHOICE_IN_SEX	1.46	1.21	2.71	2.67	1.53	1.71	1.78	1.96	1.48	1.66	1.62	1.36	1.46	2.78	1.75	1.03	1.76	1.06	1.70	1.81	1.81	1.33	1.85	3.01	1.97	4.50	0.77	0.78	0.65	0.92	0.91	1.07	1.03	9.75		2.13	3.67	3.65	3.35	2.85	3.00	1.33	4.68	4.54	2.98	0.94	0.84	0.98	0.89	0.87	0.94	0.74	1.18	1.46	0.87	0.68	0.82	1.19
q36 = MADE_FUN_OF_CALLED_NAME	2.60	0.45	1.59	1.11	0.96	0.86	0.94	0.79	1.60	1.67	1.11	0.97	1.33	1.47	1.26	1.53	2.00	1.50	1.59	1.83	1.34	1.32	1.40	1.69	2.46	1.77	0.97	0.94	1.10	1.04	1.63	0.84	1.51	2.34	2.13		8.08	12.19	7.43	1.48	1.75	1.08	2.22	1.68	1.47	0.88	0.99	1.11	0.87	0.80	0.99	0.89	1.88	1.11	0.85	1.03	0.95	1.05
q37 = PUSHED_SHOVED_TRIPPED	2.73	1.93	1.54	1.29	1.16	1.37	1.49	1.68	1.19	1.48	0.93	1.73	1.38	1.96	1.16	1.31	1.88	1.42	1.25	1.85	1.56	2.03	1.24	1.43	1.73	2.68	0.67	0.74	0.88	0.75	0.98	0.81	0.81	2.41	3.67	8.08		16.53	11.49	1.55	1.57	0.85	3.76	4.14	2.16	0.72	0.73	0.96	0.75	0.66	0.94	0.72	1.04	0.80	0.62	0.41	0.63	0.83
q38 = EXCLUDED_FROM_ACTIVITIES	1.46	1.45	1.61	1.28	1.36	1.29	1.10	1.43	1.52	1.51	0.94	1.27	1.77	2.99	1.74	1.42	2.31	1.40	1.67	1.64	1.31	1.88	1.81	1.73	2.68	2.86	0.77	0.83	0.81	0.66	0.88	0.80	1.09	2.81	3.65	12.19	16.53		9.61	1.79	1.66	1.12	3.26	2.96	2.19	0.71	0.78	1.09	0.91	0.71	0.84	0.87	1.04	1.16	0.70	0.54	0.54	1.21
q39 = SOMEONE_CALLED_YOU_GAY	1.96	1.65	1.48	1.45	0.86	0.95	0.91	1.18	1.00	1.29	1.08	1.19	1.29	1.29	1.22	1.31	2.23	1.49	1.29	2.01	1.61	1.61	1.44	1.25	1.68	2.30	0.87	0.86	1.13	1.02	1.10	0.88	1.06	2.73	3.35	7.43	11.49	9.61		2.17	1.70	1.10	2.91	3.43	2.31	0.76	0.74	0.96	0.78	0.74	0.68	0.73	0.97	1.05	0.81	0.56	0.91	0.71
q40 = ABUSED_BY_ADULT_PHYSICAL	2.03	0.84	0.94	1.59	1.33	1.21	1.33	0.83	1.30	1.06	1.25	1.03	1.24	1.53	2.49	1.09	1.39	1.18	1.18	1.65	1.38	1.48	1.26	2.28	1.87	2.08	0.98	0.88	0.93	1.16	0.95	1.20	1.05	2.44	2.85	1.48	1.55	1.79	2.17		4.61	1.93	2.03	2.57	2.04	0.58	0.58	0.91	0.78	0.59	0.63	0.77	1.74	1.35	0.90	0.85	1.11	1.97
q41 = SEEN_PARENT_ABUSED	1.19	0.23	0.92	1.35	0.77	0.93	1.56	0.90	0.99	1.18	1.07	1.11	0.90	1.91	1.32	1.14	0.94	1.57	1.31	1.60	1.71	1.56	1.34	1.56	1.37	1.55	0.80	0.93	1.23	0.80	0.83	1.10	0.82	3.71	3.00	1.75	1.57	1.66	1.70	4.61		2.80	2.06	1.57	1.48	1.04	0.90	1.06	1.01	0.99	0.85	1.02	1.10	1.29	1.06	0.95	0.99	1.35
q42 = SOMEONE_CLOSE_MURDERED	0.65	0.52	1.48	1.36	1.62	1.27	1.04	0.96	1.43	1.58	1.14	0.93	1.51	0.93	1.25	1.14	1.09	1.20	1.22	1.26	1.26	1.05	1.46	1.38	1.74	0.99	0.96	1.58	1.25	1.44	1.89	1.38	1.24	1.27	1.33	1.08	0.85	1.12	1.10	1.93	2.80		1.14	0.96	1.49	1.05	1.04	1.21	1.05	1.20	1.09	1.05	1.12	1.84	1.15	1.67	1.03	1.70
q43 = DAYS_YOU_SMOKE_CIGS	1.25	1.75	1.59	1.87	1.34	1.86	1.76	1.68	1.79	2.42	1.74	2.16	1.93	1.59	2.36	1.07	1.24	1.16	1.41	1.10	1.27	1.31	1.79	1.80	1.96	3.40	0.50	0.50	0.60	0.70	0.58	0.84	0.74	1.89	4.68	2.22	3.76	3.26	2.91	2.03	2.06	1.14		26.83	16.08	0.54	0.46	0.51	0.48	0.52	0.53	0.51	1.21	1.22	1.20	0.40	0.51	1.13
q44 = DAYS_DRINK_ALC	1.73	2.98	3.59	1.84	1.49	2.35	2.01	2.05	1.86	1.69	1.76	2.26	1.93	1.44	2.16	1.03	1.74	1.17	1.45	1.20	1.51	1.37	1.59	1.29	2.14	2.60	0.55	0.46	0.53	0.59	0.40	0.67	0.84	2.25	4.54	1.68	4.14	2.96	3.43	2.57	1.57	0.96	26.83		26.31	0.46	0.44	0.63	0.50	0.49	0.51	0.57	1.33	0.66	1.00	0.29	0.50	0.58
q45 = DAYS_USE_MARIJUANA	1.26	1.97	2.69	2.28	1.86	2.23	1.99	1.88	1.93	2.28	2.26	2.12	2.04	1.80	2.52	1.11	1.33	1.47	1.34	1.15	1.39	1.68	2.14	1.75	1.56	3.40	0.68	0.57	0.67	0.72	0.54	0.99	0.77	1.85	2.98	1.47	2.16	2.19	2.31	2.04	1.48	1.49	16.08	26.31		0.54	0.51	0.65	0.51	0.66	0.56	0.69	1.12	1.35	1.32	0.64	0.55	1.09
q46 = CLOSE_TO_PEOPLE_AT_SCHOOL	0.75	0.64	0.26	0.95	0.84	0.96	1.19	1.42	0.88	1.49	0.95	1.06	0.84	0.79	0.42	1.26	1.11	0.83	1.29	0.95	1.04	0.76	0.85	0.76	0.75	0.60	1.65	2.00	2.53	1.85	1.85	1.75	1.59	0.85	0.94	0.88	0.72	0.71	0.76	0.58	1.04	1.05	0.54	0.46	0.54		16.64	8.68	8.93	10.42	6.12	10.57	0.85	0.90	0.91	1.64	2.12	2.46
q47 = FEEL_HAPPY_AT_SCHOOL	0.55	0.45	0.55	0.74	0.71	1.02	0.92	1.07	0.86	1.06	0.78	0.82	0.86	0.77	0.52	1.14	1.14	0.80	1.33	1.01	0.71	0.71	0.71	0.77	0.75	0.60	1.81	1.96	2.46	1.69	1.92	1.80	1.74	0.77	0.84	0.99	0.73	0.78	0.74	0.58	0.90	1.04	0.46	0.44	0.51	16.64		12.93	10.27	19.46	10.27	11.65	0.81	0.81	1.32	1.78	1.93	2.44
q48 = SCHOOL_SUPORTIVE_LEARNING	0.84	0.58	0.41	0.76	0.90	0.84	1.01	1.20	0.64	1.07	0.87	0.76	0.80	1.19	0.80	1.04	1.36	0.97	1.31	0.96	0.89	0.80	0.82	0.75	0.81	0.85	1.83	2.56	2.60	1.97	2.22	1.55	2.13	0.93	0.98	1.11	0.96	1.09	0.96	0.91	1.06	1.21	0.51	0.63	0.65	8.68	12.93		31.37	19.05	13.53	19.20	1.00	0.98	1.20	2.20	1.87	2.94
q49 = SCHOOL_PROMOTE_DIVERSITY	0.78	0.24	0.24	0.70	0.71	0.67	0.92	0.90	0.72	1.00	0.85	0.71	0.80	1.33	0.71	1.12	1.02	0.81	1.05	0.96	0.82	0.93	0.89	0.71	0.68	0.79	1.52	1.87	2.11	1.51	1.34	1.51	1.24	0.86	0.89	0.87	0.75	0.91	0.78	0.78	1.01	1.05	0.48	0.50	0.51	8.93	10.27	31.37		15.34	13.46	13.09	0.70	0.94	1.18	1.30	1.46	2.41
q50 = FEEL_PART_OF_SCHOOL	0.80	0.27	0.49	0.69	0.60	0.69	0.89	1.04	0.63	0.98	1.00	0.62	0.72	1.14	0.89	1.18	1.10	1.12	1.29	1.10	0.90	0.80	0.94	0.80	0.68	0.84	1.53	2.48	2.56	1.96	1.67	1.77	1.80	0.71	0.87	0.80	0.66	0.71	0.74	0.59	0.99	1.20	0.52	0.49	0.66	10.42	19.46	19.05	15.34		12.02	11.64	0.92	0.84	1.57	1.94	1.71	2.34
q51 = TREATED_FAIRLY_BY_TEACHERS	1.11	0.45	0.80	0.61	0.66	0.84	1.02	0.88	0.85	0.87	0.98	0.86	0.81	0.82	0.68	0.60	0.74	0.65	0.89	0.78	0.56	0.65	0.63	0.65	0.61	0.56	1.26	1.81	2.10	1.87	1.88	1.59	1.56	0.88	0.94	0.99	0.94	0.84	0.68	0.63	0.85	1.09	0.53	0.51	0.56	6.12	10.27	13.53	13.46	12.02		16.02	0.79	0.89	1.65	1.98	1.57	3.23
q52 = SCHOOL_IS_SAFE	0.82	1.13	0.55	0.93	0.87	0.97	1.13	1.18	0.94	1.04	1.11	1.02	0.73	0.65	0.65	0.86	1.01	0.80	1.17	0.65	0.75	0.77	0.69	0.75	0.68	0.70	1.56	2.36	2.26	1.57	2.03	1.54	1.76	1.17	0.74	0.89	0.72	0.87	0.73	0.77	1.02	1.05	0.51	0.57	0.69	10.57	11.65	19.20	13.09	11.64	16.02		0.73	0.90	1.15	1.60	1.94	2.72
q53 = FRIENDS_GET_IN_TROUBLE	0.47	1.12	0.92	0.89	1.21	1.09	0.72	0.60	0.98	0.79	0.68	0.71	0.71	0.77	2.06	1.02	1.51	1.57	1.53	1.64	1.80	1.17	1.54	1.47	1.80	1.86	1.17	0.90	0.92	0.98	1.02	0.71	0.91	1.44	1.18	1.88	1.04	1.04	0.97	1.74	1.10	1.12	1.21	1.33	1.12	0.85	0.81	1.00	0.70	0.92	0.79	0.73		1.24	0.94	1.02	0.76	1.03
q54 = BEEN_SUSPENDED	0.91	0.72	0.48	1.49	1.66	1.04	0.82	0.83	1.17	1.56	1.25	0.87	1.20	1.34	1.11	0.97	0.98	1.32	1.40	1.20	1.29	1.30	3.97	1.87	1.66	1.26	1.21	1.19	1.01	1.01	1.34	0.78	0.83	1.45	1.46	1.11	0.80	1.16	1.05	1.35	1.29	1.84	1.22	0.66	1.35	0.90	0.81	0.98	0.94	0.84	0.89	0.90	1.24		1.08	1.96	0.67	1.10
q55 = HAVE_SKILLS_FOR_JOB	0.94	0.55	0.87	0.75	0.74	0.52	0.48	0.51	0.95	1.07	0.88	0.55	1.08	0.32	0.57	1.21	0.70	1.01	0.87	1.00	0.70	0.81	1.23	0.73	0.58	0.88	2.63	3.16	2.89	3.42	3.92	3.23	2.60	0.92	0.87	0.85	0.62	0.70	0.81	0.90	1.06	1.15	1.20	1.00	1.32	0.91	1.32	1.20	1.18	1.57	1.65	1.15	0.94	1.08		9.80	4.35	2.73
q56 = IMPORTANT_TO_HAVE_JOB	0.78	0.25	0.37	0.77	0.94	0.50	0.55	0.53	0.92	1.57	1.40	0.58	1.05	0.18	0.35	0.80	0.37	0.59	0.56	0.85	0.54	0.58	1.10	0.55	0.35	0.30	8.59	9.11	9.48	9.24	11.86	7.50	5.87	1.27	0.68	1.03	0.41	0.54	0.56	0.85	0.95	1.67	0.40	0.29	0.64	1.64	1.78	2.20	1.30	1.94	1.98	1.60	1.02	1.96	9.80		7.80	3.87
q57 = EARNING_GOOD_LIVING	0.84	0.36	0.53	0.67	0.82	0.80	0.68	0.59	0.85	1.17	0.99	0.71	0.85	0.27	0.55	0.86	0.86	0.76	0.74	0.63	0.80	0.62	0.83	0.72	0.66	1.05	3.98	5.75	5.94	5.04	4.60	4.71	5.37	2.06	0.82	0.95	0.63	0.54	0.91	1.11	0.99	1.03	0.51	0.50	0.55	2.12	1.93	1.87	1.46	1.71	1.57	1.94	0.76	0.67	4.35	7.80		2.42
q58 = ARE_YOU_IN_SCHOOL	0.50	0.27	0.52	0.83	1.01	0.83	0.75	0.74	1.24	1.48	1.08	0.95	0.99	0.43	0.31	0.99	0.82	0.98	1.17	0.81	0.93	0.98	1.02	1.15	0.63	0.42	1.28	2.01	2.02	2.03	2.78	2.16	2.11	1.02	1.19	1.05	0.83	1.21	0.71	1.97	1.35	1.70	1.13	0.58	1.09	2.46	2.44	2.94	2.41	2.34	3.23	2.72	1.03	1.10	2.73	3.87	2.42	

(pink)	OR >=10
(orange)	OR 3-9.9
(light yellow)	OR 2-2.9
(green)	OR <0.5

© 2019 Culyba AJ et al. JAMA Network Open.

eFigure 1. Co-occurrence of Risk and Protective Behaviors Among Youth With Natural Mentoring

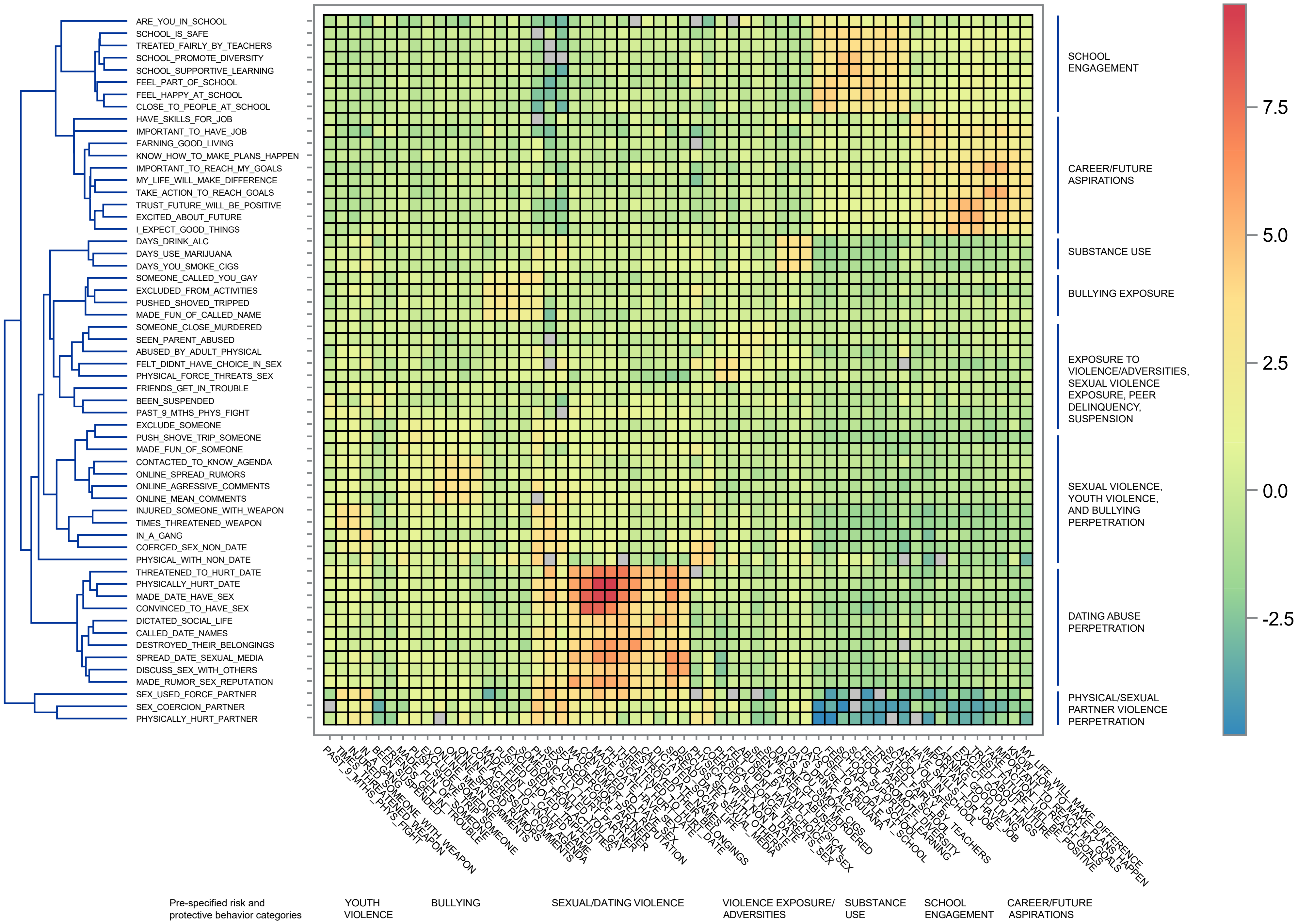

Dendrogram and heat map derived from the odds ratio matrix across 58 risk and protective behaviors among participants with natural mentoring. The 3364 crude odds ratios were normalized and hierarchical clustering was used to define co-occurrence patterns. The x-axis represents the prespecified domains and the y-axis represents the clustering of behaviors among participants with natural mentoring.

© 2019 Culyba AJ et al. JAMA Network Open.

eFigure 2. Co-occurrence of Risk and Protective Behaviors Among Youth Without Natural Mentoring

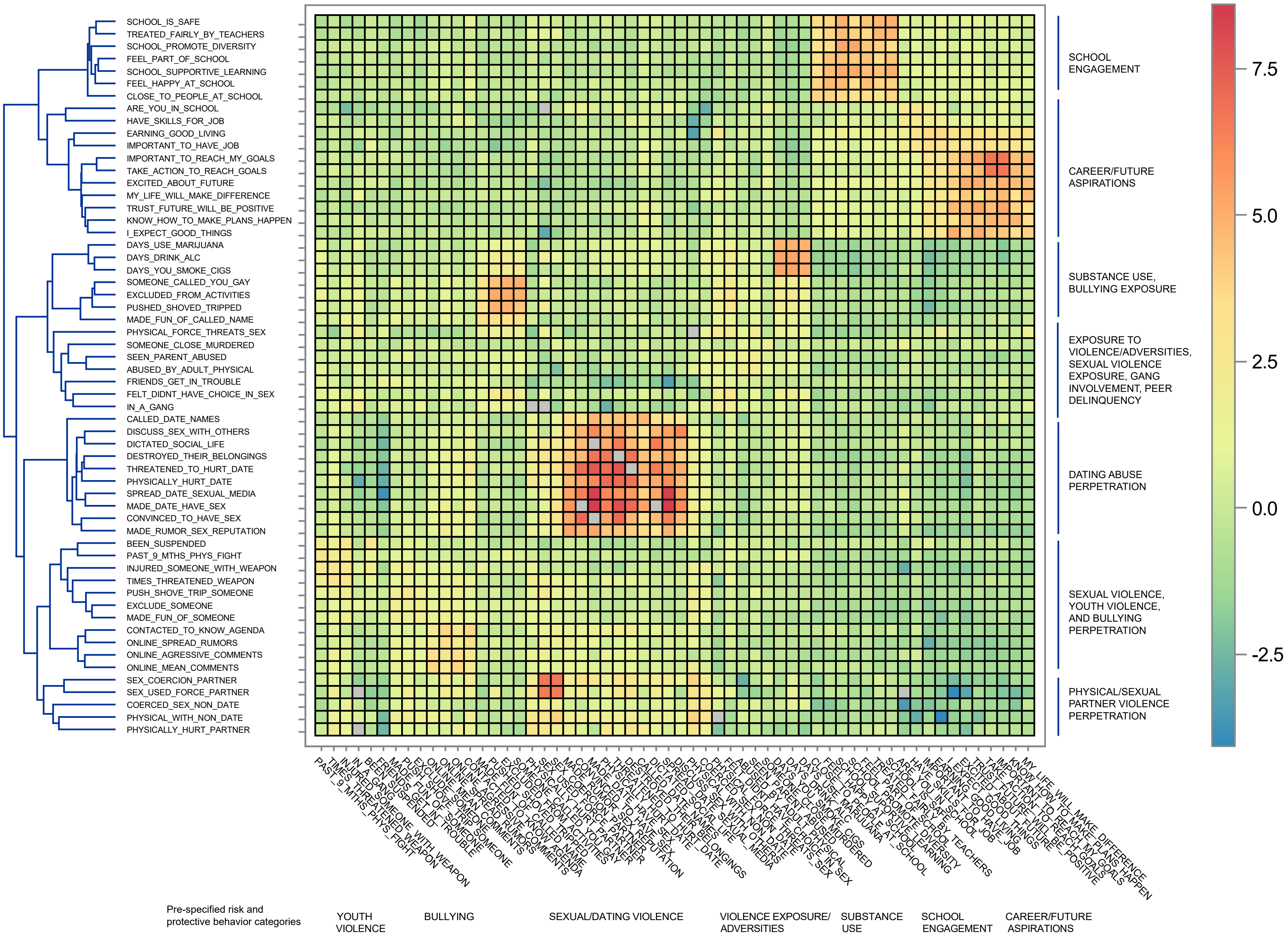

Dendrogram and heat map derived from the odds ratio matrix across 58 risk and protective behaviors among participants without natural mentoring. The 3364 crude odds ratios were normalized and hierarchical clustering was used to define co-occurrence patterns. The x-axis represents the prespecified domains and the y-axis represents the clustering of behaviors among participants without natural mentoring.

© 2019 Culyba AJ et al. JAMA Network Open.
